# Optical Coherence Tomography Angiography Changes in Patients Diagnosed With Acute Coronary Syndrome: A Systematic Review and Meta-Analysis

**DOI:** 10.7759/cureus.54121

**Published:** 2024-02-13

**Authors:** Anna Maria Sideri, Dimitra Mitsopoulou, Stylianos A Kandarakis, Andreas Katsimpris, Menelaos Kanakis, Aristotelis Karamaounas, Dimitrios Brouzas, Petros Petrou, Evangelia Papakonstantinou, Konstantinos Droutsas, Georgios Giannopoulos, Ilias Georgalas

**Affiliations:** 1 First Department of Ophthalmology, G. Gennimatas Hospital, National and Kapodistrian University of Athens, Athens, GRC; 2 Ophthalmology, Princess Alexandra Eye Pavilion, Edinburgh, GBR; 3 Ophthalmology, University Eye Clinic, Rion University Hospital, University of Patras, Patras, GRC; 4 Ophthalmology, National and Kapodistrian University of Athens, Athens, GRC; 5 Third Department of Cardiology, School of Medicine, Aristotle University of Thessaloniki, Thessaloniki, GRC

**Keywords:** optical coherence tomography angiography (oct-a), st-segment elevation myocardial infarction (stemi), acs (acute coronary syndrome), deep vascular plexus, superficial vascular plexus, foveal avascular zone

## Abstract

We conducted a systematic review and meta-analysis to assess the association between optical coherence tomography angiography (OCTA) parameters and acute coronary syndrome (ACS). Two independent reviewers searched the electronic databases (MEDLINE (Medical Literature Analysis and Retrieval System Online), Scopus, Embase (Excerpta Medica Database), Cochrane Library, ClinicalTrials.gov, and World Health Organization International Clinical Trials Registry Platform) from inception until April 2023. According to the inclusion criteria of this review, eligible were observational studies, randomized control trials, and registry/database studies that included the eyes of adult ACS patients and assessed OCTA parameters within the macula. The pooled standardized mean differences (SMD) between patients diagnosed with ACS and healthy controls with a confidence interval (CI) of 95% were calculated using the Hartung-Knapp-Sidik-Jonkman random-effects method. The heterogeneity was assessed by I^2^ and the Cochran Q and a random effects model was applied. Seven studies were eligible and included in our systematic review (n = 898), of which three were included in the meta-analysis (n = 341). The pooled SMD in the superficial vascular plexus (SVP), deep vascular plexus (DVP), and foveal avascular zone (FAZ) were -0.46 (95% CI: -0.94 to 0.01, p = 0.05, I^2^ = 0%, three studies), -0.10 (95% CI: -3.20 to 3.00, p = 0.75, I^2^ = 67%, two studies), and 0.43 (95% CI: -1.22 to 2.09, p = 0.38, I^2^ = 92%, three studies), respectively. Our findings suggest that there are no differences in OCTA metrics between ACS patients and healthy individuals.

## Introduction and background

Coronary heart disease (CHD) or coronary artery disease (CAD) is the most common type of cardiovascular disease (CVD) and approximately 610,000 deaths are reported each year from it in the United States. Diabetes mellitus (DM), hypertension, hypercholesterolemia, and smoking have been recognized as risk factors [1,2].

Acute coronary syndrome (ACS) refers to myocardial ischemia due to insufficient coronary blood flow and may be presented as ST-elevation myocardial infarction (STEMI), non-ST elevation myocardial infarction (NSTEMI), and unstable angina. According to a recent meta-analysis, the global prevalence of myocardial infarction (MI) in individuals under or over 60 years old is estimated at around 3.8% and 9.5%, respectively [3]. The prognostic values of coronary microvascular dysfunction (CMD) and post-ischemic CMD have been delineated, highlighting the microvascular component in ACS [4,5].

The retinal vasculature shows some anatomical and physiological similarities with that of the brain, the kidney, and the heart [6]. Changes in retinal vascular structure have shown a correlation with CAD and the severity of the disease [7]. The introduction of optical coherence tomography angiography (OCTA), a non-invasive imaging modality, allows direct in-vivo visualization and assessment of retinal microvasculature [8]. The foveal avascular zone (FAZ), the superficial and deep microvasculature, the choriocapillaris (CC), and other parameters have been quantitively assessed in patients with CVD [9-12].

Whether retinal microvascular morphology reflects coronary microcirculation and whether OCTA biomarkers could be used for CMD in CHD seem to be drawing intense attention. Therefore, the aim of the present systematic review and meta-analysis is to identify and quantify retinal microvascular changes detected by OCTA in patients with STEMI.

## Review

Materials and methods

Eligibility Criteria

This systematic review and meta-analysis was conducted based on PRISMA (Preferred Reporting Items for Systematic Reviews and Meta-Analyses) guidelines [13]. A protocol for this review was designed and prepared using the PICO (population, intervention, control, and outcomes) framework. However, it was not registered. No amendments to the protocol were required. Inclusion criteria for the studies were: (i) observational studies, randomized clinical trials (RCTs), and registry/database studies; (ii) patients diagnosed with ACS; and (iii) retinal microvasculature measurements within the macula using OCTA. Reviews, meta-analyses, case reports, conference abstracts or presentations, non-human subject research, and articles not in English were excluded. Studies that involved patients with ACS without further defining its type were also excluded.

Literature Search and Study Selection

MEDLINE (Medical Literature Analysis and Retrieval System Online), Scopus, Embase (Excerpta Medica Database), Cochrane Library, ClinicalTrials.gov, and the World Health Organization International Clinical Trials Registry Platform (WHO ICTRP) were searched (up to April 20, 2023). The search strategy used in each database and registry is shown in Table 1. Snowball search method was also conducted manually, in order not to miss eligible studies. The systematic literature search was performed by two authors (AMS, DM) independently. Duplicates were removed by EndNote (Clarivate PLC, London, United Kingdom). Disagreements were resolved by consensus after discussion. All studies were compared to avoid overlapping populations; in such a case, the study with the largest sample was included.

The eligibility screening step was performed by the same two investigators (AMS, DM) independently, based on prespecified criteria. Studies that did not meet the latter were considered ineligible and were excluded. Consensus after discussion was reached in the case of conflicts between the two authors.

Data Extraction

Two authors (AMS, DM) independently performed data extraction on a predefined customized form, which included the first author’s name and year of publication, study design, sample size and number of eyes included in the study, mean age, sex, OCTA machine type used, macular scan diameter, signal strength threshold, and OCTA parameters assessed in each study. Exclusion criteria defined by each study were also extracted. For any additional information and explanation regarding the studies, the authors were contacted.

Risk of Bias Assessment

An adapted version of the Newcastle-Ottawa Scale (NOS) for the included studies was used for the risk of bias assessment. It is a seven-item scale divided into three categorical criteria: selection of study groups, their comparability and assessment of the outcome of interest, and the statistical test [14]. A study of the highest quality can be awarded a maximum of 10 points; of low risk of bias were the studies that collected nine or 10 points, studies with a total score of seven or eight points were considered of medium risk of bias, while those that scored less than six points were considered of high risk of bias. The two authors (AMS, DM) independently performed the assessment and graded the quality of the studies. Discrepancies were resolved by consensus.

Statistical Analysis

We utilized the means and standard deviations extracted from each outcome group to compute standardized mean differences (SMDs) for each OCTA metric across distinct outcome groups, accompanied by corresponding 95% confidence intervals (95% CIs). In instances where direct OCTA measurements were not available, values were indirectly calculated through a combination of means and standard deviations. We utilized the Hartung-Knapp/Sidik-Jonkman random-effects method to meta-analyze study-specific SMDs and produce pooled effect estimates along with their respective 95% CIs, while also estimating the variance between studies (τ^2^). The Hartung-Knapp/Sidik-Jonkman method offers notable advantages, particularly in cases of substantial heterogeneity among studies or when the number of studies in the meta-analysis is limited, as discussed previously [15,16]. Moreover, heterogeneity's impact in our pooled estimate was further investigated through the calculation of the percentage of total variation attributable to heterogeneity (I^2^), and the assessment of heterogeneity between studies was conducted using the Cochran Q test. Given the comparatively limited number of eligible studies, we refrained from conducting tests to assess publication bias or utilizing meta-regression to identify potential sources of heterogeneity.

**Table 1:** Search strategies for all databases and registers ICTRP: International Clinical Trials Registry Platform

Database	Search strategy
PubMed	((“OCTA”) OR (“OCT-A”) OR (“OCT-angio”) OR (“angio-OCT”)) AND ("acute myocardial infarction" OR "acute coronary syndrome" OR “STEMI” OR “heart”)
Scopus	#1 (“OCTA”) OR (“OCT-A”) OR (“OCT-angio”) OR (“angio-OCT”) in Article title, Abstract, Keywords #2 ("acute myocardial infarction" OR "acute coronary syndrome" OR “STEMI” OR “heart”) in Article title, Abstract, Keywords Combined: #1 AND #2
Embase	#1 (‘OCTA’) OR (‘OCT-A’) OR (‘OCT-angio’) OR (‘angio-OCT’): ti,ab,kf #2 (‘acute myocardial infarction’ OR ‘acute coronary syndrome’ OR ‘STEMI’ OR ‘heart’): ti,ab,kf Combined: #1 AND #2
Cochrane Library	#1 (‘OCTA’) OR (‘OCT-A’) OR (‘OCT-angio’) OR (‘angio-OCT’): ti,ab,kw #2 (‘acute myocardial infarction’ OR ‘acute coronary syndrome’ OR ‘STEMI’ OR ‘heart’): ti,ab,kw Combined: #1 AND #2
WHO ICTRP	Condition: STEMI OR ST Elevation Myocardial Infarction OR acute coronary syndrome Intervention: Optical coherence tomography angiography OR OCTA
ClinicalTrials.gov	Condition/Disease: STEMI - ST Elevation Myocardial Infarction Intervention/treatment: Optical coherence tomography angiography OCTA

Results

Systematic review

**Figure 1:**
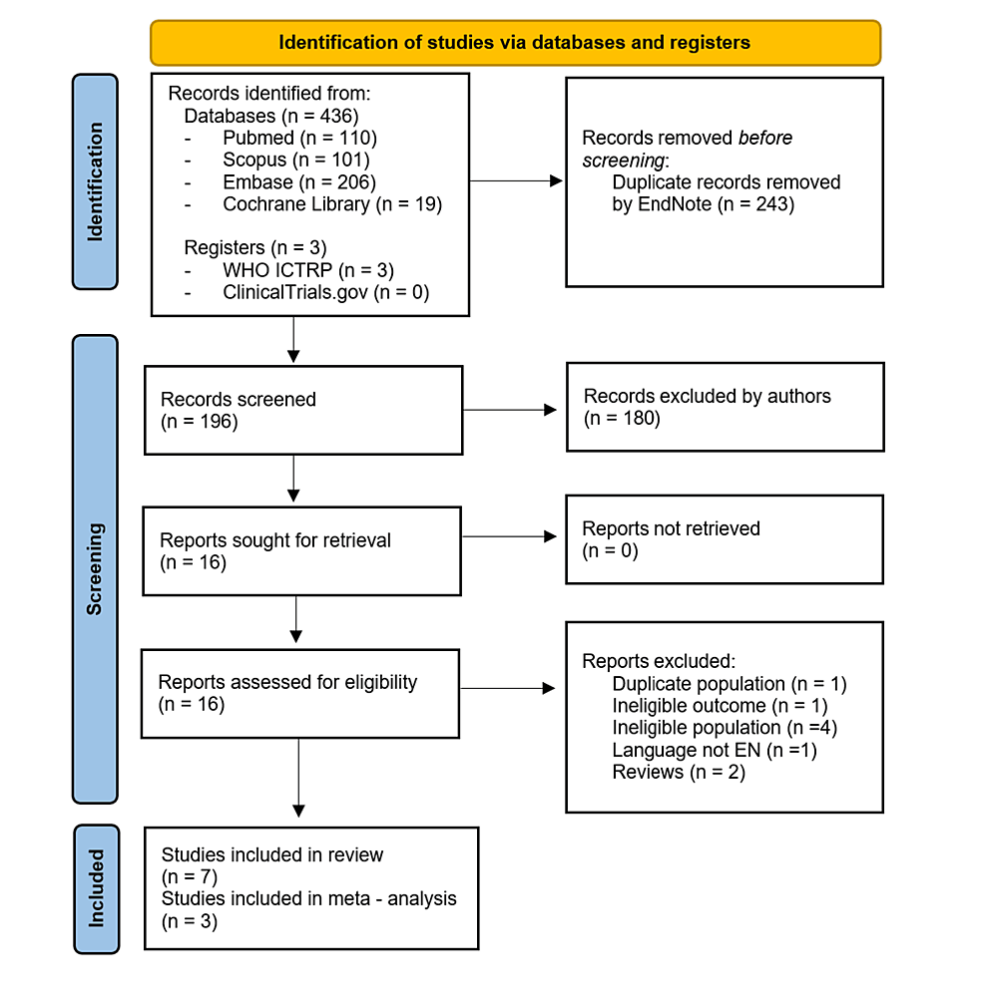
PRISMA flow diagram of the study selection process PRISMA: Preferred Reporting Items for Systematic Reviews and Meta-Analyses; ICTRP: International Clinical Trials Registry Platform EndNote (Clarivate PLC, London, United Kingdom)

Study selection: Our literature search yielded 439 studies in total. After removing duplicates, 196 records were screened by title and abstract, and 16 full-text articles were assessed for eligibility. In one study [15], the participants were a subgroup of a larger study [16]; the former study was excluded in order to avoid duplicate populations and the latter was included. One non-English publication [17] and two reviews [10,12] were also excluded from the systematic review. One study used OCTA measurements to associate acute kidney injury with low retinal vascular density in patients with ACS [18] and was also excluded. Studies that included patients with CAD and did not specify the presence of ACS were considered ineligible and excluded from the systematic review and the meta-analysis [9,19-21]. Studies that did not use a control group were not included in the meta-analysis [16,22,23] Thus, seven studies were eligible for the systematic review [11,16,22-26], three of which were also included in the meta-analysis. The screening process is summarized in the Preferred Reporting Items for Systematic Reviews and Meta-Analyses (PRISMA) flow chart in Figure 1.

Study characteristics: All the eligible studies were cross-sectional. In total, 898 participants were included in the systematic review (660 ACS patients, 238 healthy participants) and 341 (185 in the ACS group, 156 in the control group) in the analysis. The mean age of the ACS patients ranged from 55.9 to 63 years old and the majority were male patients; the female sex ratio ranged from 4.9% to 21.5% in all studies except one [24], in which 77% of patients were females. Regarding the type of ACS, only one study categorized the patients further into the three subgroups of ACS (STEMI, NSTEMI, unstable angina) [16], whilst the rest of the studies did not include [11,22,23] or specify [24-26] a subgroup of patients with unstable angina. Two studies included NSTEMI patients [16,22] and one study included only patients diagnosed with STEMI [11].

In two studies, OCTA measurements were conducted using the Cirrus HD-OCT, Model 5000 (Carl Zeiss AG, Oberkochen, Baden-Wurttemberg, Germany) with Cirrus AngioPlex software, version 10, software [16,25], while two studies used the AngioVue Avanti with RTVue-XR, version 2017.1.0.155, software (Optovue Inc., Fremont, California, United States) [24,26], and three studies used the DRI Optical Coherence Tomography (OCT) Triton (Topcon Corporation, Tokyo, Japan) [11,22,23] with Topcon's IMAGEnet 6, version 1.14, [11], Fiji, version 1.53f51, IMAGEnet 6, version 1.28.17646, software [22]. The time at which OCTA was performed was vastly different among studies and varied from 48 hours within admission to seven days post coronary angiography.

Furthermore, the method of eye selection differed among the selected studies. More specifically, in three studies, OCTA was performed on both eyes, but only one eye was selected for the analysis [11,7,25]: the functional eye for single-eye patients [16,25], the right eye for an even-numbered year of birth of the participants, the left eye for an odd-numbered year of birth, and the exclusion of the eye with uninterpretable scan [16,26] or worse quality of acquisition [25]. In another study, unremarkable media opacity was a prerequisite, and the eye with the best-corrected visual acuity (BCVA) was preferred [22], whereas one study assessed both eyes in case of adequate scan quality and in the absence of artifacts [11]. Two studies did not report whether one or both eyes were included in the analysis [23,24]. The signal strength threshold was 7/10 for two studies [16,25], and the image quality index was 6/10 for another study [26]. One study set OCT signal strength index < 45 and OCTA image quality < 60 [22], while another one defined signal strength index ≥ 60 and scan quality index ≥ 8 [24]. One study set scan quality > 40 [11]. Two studies did not report a signal strength threshold [23,24]. The characteristics of the included studies are described in Table 2.

**Table 2:** Main characteristics of the included studies NOS: Newcastle–Ottawa Scale; NR: not reported; NSTEMI: non-ST elevation myocardial infarction; STEMI: ST-elevation myocardial infarction

Author and year	Study design	Patients/eyes, n	Age (mean ± SD)	Female sex, n (%)	Axial length (mean ± SD)	STEMI, n (%)	NSTEMI, n (%)	Unstable angina, n (%)	NOS score
Arnould et al. 2018 [16]	Cross-sectional	237/237	62.0 ± 13.0	51 (21.5)	23.43 ± 0.96	94 (39.7)	113 (47.6)	30 (12.7)	8/10
Ay et al. 2023 [24]	Cross-sectional	35/NR	61.00 ± 10.00	27 (77)	NR	NR	NR	NR	6/10
Hannappe et al. 2020 [25]	Cross-sectional	62/62	63 ± 3.4411	12 (19)	23.75 ± 0.16	40 (65)	NR	NR	7/10
Kim et al. 2022 [22]	Cross-sectional	60/60	59 ± 12	6 (10)	NR	37 (61.7)	23 (38.3)	0	8/10
Matuleviciute et al. 2022 [23]	Cross-sectional	76	61.29 ± 8.56	28 (37)	NR	NR	NR	0	9/10
Sideri et al. 2023 [11]	Cross-sectional	88/176	55.9 ± 13.7	5 (5.7)	NR	88 (100)	0	0	10/10
Zhong et al. 2021 [26]	Cross-sectional	102/102	58.83 ± 8.9	5 (4.9)	NR	NR	NR	NR	8/10

Table 3 summarizes the methodological characteristics and outcomes of the included studies.

**Table 3:** Methodological characteristics and outcomes of the included studies OCTA: optical coherence tomography angiography; OCT: optical coherence tomography; ACS: acute coronary syndrome; AL: axial length; AMI: acute myocardial infarction; CC: choriocapillaris; CSC: chronic coronary syndrome; CTO: coronary total occlusion; D: diopters; DCP: deep capillary plexus; ERM: epiretinal membrane; FAZ: foveal avascular zone (perimeter in mm, area in mm2); GCL+:  ganglion cell and inner plexiform layer; IOP: intraocular pressure; MH: macular hole; NR: not reported; OD: optic disc; PCI: percutaneous coronary intervention; RNFL: retinal nerve fiber layer; RPC: radial peripapillary capillary; SCP: superficial capillary plexus (% or mm-1); VD: vessel density (%)

Author and year	Inclusion criteria	Exclusion criteria	OCTA machine	Macular scan diameter (mm)	Time at which OCTA performed	OCTA parameters
Arnould et al. 2018 [16]	Patients with ACS, with or without ST-segment elevation	i) preexisting retinal disease, ii) age < 18 years of age, iii) severe myopia/ AL > 26 mm, iv) hemodynamic instability, v) refusal to participate, vi) no national health insurance, vii) guardianship	CIRRUS HD-OCT, Model 5000 (Carl Zeiss AG, Oberkochen, Baden-Wurttemberg, Germany)	3x3	Within 48 hours of admission	FAZ, perfusion density, and SCP VD in different quadrants
Ay et al. 2023 [24]	Patients with ACS and CSC, based on coronary angiography results; control group: healthy participants	i) preexisting ocular pathology (macular edema, corneal, or lens opacity), ii) history of ocular surgery and/or trauma, iii) recent use of eye lubricants or contact lenses, iv) IOP ≥ 21 mmHg, v) refractive error >±1.5 D, vi) AL > 26.5 mm, vii) disability to cooperate with OCTA procedure, viii) inability to participate due to CAD severity, ix), x) history of any malignancy or Behçet’s disease, xi) regular alcohol consumption	AngioVue Avanti RTVue-XR (Optovue Inc., Fremont, California, United States)	6x6	Three to seven days post coronary angiography	CC, FAZ, DCP VD, SCP VD, and OD RNFL thickness and VD
Hannappe et al. 2020 [25]	Patients with ACS, with or without ST-segment elevation; control group: patients >40 years old without cardiovascular risk	i) preexisting retinal disease or glaucoma ii) AL > 26 mm, iii) hemodynamic instability, iv) refusal to participate, v) no national health insurance, vi) guardianship	CIRRUS HD-OCT, Model 5000	3x3	Within 48 hours of the cardiovascular event	FAZ, perfusion density, and SCP VD
Kim et al. 2022 [22]	Patients with AMI, with or without ST-segment elevation	i) preexisting retinal disease or glaucoma, ii) history of intraocular surgery or intravitreal injection, iii) refractive error > ±2.0 D, iv) OCT signal strength index < 45 and OCTA image quality < 60, v) refusal of examination, vi) prior revascularization, vii) atrial fibrillation, vii) end-stage renal disease,	DRI OCT Triton (Topcon Corporation, Tokyo, Japan)	4.5x4.5	Within 72 hours of admission	CC, DCP VD, and SCP VD
Matuleviciute et al. 2022 [23]	Patient group with AMI, with or without ST-segment elevation, and patient group with three-vessel disease; control group: healthy participants with unobstructed coronary arteries	i) preexisting retinal disease or any conditions obscuring the view of the fundus, ii) history of ocular surgery (uneventful phacoemulsification excluded) or trauma, iii) amblyopia, iv) intraocular inflammation, v) glaucoma, vi) refractive error > ±6.0 D (myopia and hyperopia) or > ± 3.0 D (astigmatism), vii) diabetes mellitus	DRI OCT Triton	3x3, 6x6	Within five days of the cardiovascular event	Choroidal thickness, DCP VD, SCP VD, RNFL thickness, and GCL+ layer thickness
Sideri et al. 2023 [11]	Hemodynamically stable patients with STEMI; control group: healthy participants > 30 years old	i) preexisting retinal disease, intraocular hypertension, or glaucoma, ii) history of ocular inflammation or trauma, iii) history of vitrectomy, iv) presence of ERM or MH, v) refractive error ≥±3 D	DRI OCT Triton	3x3	Within 48 hours of admission	CC, DCP VD, FAZ, and SCP VD
Zhong et al. 2021 [26]	Patients with ≥ 1 CTO and CAD without CTO, hospitalized for PCI	i) preexisting hypertensive or diabetic retinopathy, ii) refractive error ≥ ±3 D, iii) disability to cooperate with ophthalmic examinations, iv) hemodynamic instability, v) prior revascularization	AngioVue Avanti RTVue-XR	6x6	NR	Macular VD, DCP VD, SCP VD, RNFL thickness, and RPC density

Risk of bias in studies: The modified NOS scale adapted for cross-sectional studies was used. The majority of the studies were characterized as having medium risk of bias [16,22,25,26]. One study was awarded six stars and considered to have a high risk of bias [26], while one study was marked with the maximum, i.e., 10 stars [11]. Two studies were not given stars for comparability [24,25], while only two studies justified the size of the sample [11,23]. The risk of bias in the included studies is presented in Table 4.

**Table 4:** Risk of bias assessment of the included studies, using the modified Newcastle-Ottawa Scale (mNOS) for cross-sectional studies - = o; * = 1; ** = 2 1. Selection of participants: a) Representativeness of the sample (1 = participants were randomly selected or consecutive eligible participants were selected, or all participants were invited to participate from the source population, 0 = selected group or no explanation). b) Sample Size (1 = justified and satisfactory, 0 = not justified). c) Response rate/non-responders (number of participants was recorded compared with the number of participants intended to be recorded in the study design, or the number of participants that fulfilled study requirements) (1 = response rate > 80%, 0 = not). d) Ascertainment of exposure (2 = optical coherence tomography angiography (OCTA) microvascular parameters automatically measured by software, 1 = OCTA microvascular parameters not automatically measured by software, 0 = not explained). 2. Comparability (confounding factors are checked, and there is comparability between subject groups) (2 = more than one factor checked, 1 = one major factor checked, 0 = no factors checked). 3. Outcome. a) Assessment of outcome (2 = independent blind assessment, reference to secure records or record linkage, 1 = self-report, 0 = no description). b) Statistical analysis (1 = statistical analysis adequate, tools described, 0 = not adequate, no description)

Author and year	Selection				Comparability	Outcome		Score
	Representativeness of the sample	Sample size justified	Non-respondents	Ascertainment of exposure	Confounding controlled	Outcome assessment	Statistical test	Total
Arnould et al. 2018 [16]	*	-	-	**	**	**	*	8/10
Ay et al. 2023 [24]	*	-	-	**	-	**	*	6/10
Hanappe et al. 2020 [25]	*	-	*	**	-	**	*	7/10
Kim et al. 2022 [22]	*	-	-	**	**	**	*	8/10
Matuleviciute et al. 2022 [23]	*	*	-	**	**	**	*	9/10
Sideri et al. 2023 [11]	*	*	*	**	**	**	*	10/10
Zhong et al. 2021 [26]	*	-	-	**	**	**	*	8/10

Meta-Analysis

**Figure 2:**
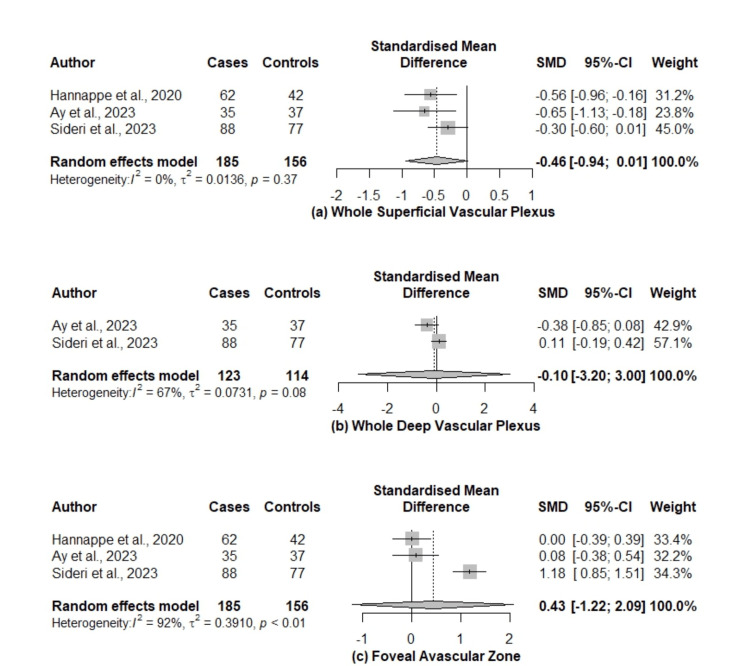
Forest plots of the SMDs on patients with ACS and healthy participants ACS: acute coronary syndrome; CI: confidence interval; SMDs: standardized mean differences [11,24,25]

In our study, we used the SMD as a summary statistic in order to analyze the OCTA metrics obtained from the eligible studies, as different OCT machines were used. The SMDs between superficial capillary plexus (SCP), deep capillary plexus (DCP), and the FAZ of ACS patients and healthy controls were pooled. A vast heterogeneity across the studies assessing DCP and FAZ was observed. The pooled SMDs regarding meta-analyzed OCTA metrics between ACS patients and healthy participants are presented in Figure 2.

Whole superficial vascular plexus: Data from three studies (n =341) were used to assess the density of the SCP [11,24,25]. The pooled SMD between ACS patients and healthy controls was -0.46 (95% CI: -0.94 to 0.01, p = 0.05, I^2^ = 0%), presenting marginally non-significant lower values for the ACS group. However, in one study, the SVP did not significantly differ between the two groups [11].

Whole deep vascular plexus: Estimates were obtained from two studies (n = 237) [11,24]. The density of the DCP was reduced in ACS patients compared to the control group, but the difference was not significant; the pooled SMD was -0.10 (95% CI: -3.20 to 3.00, p = 0.75, I^2^ = 67%).

FAZ: The FAZ metrics were evaluated in three studies [11,24,25] and the results of the analysis gave a pooled SMD of 0.43 (95% CI: -1.22 to 2.09, p = 0.38, I^2^ = 92%) between the two groups indicating no difference. Nevertheless, Sideri et al. [11] showed a significant difference in FAZ size in patients diagnosed with STEMI (p < 0.001), with respect to the number of affected coronary arteries.

Discussion

In this study, we systematically reviewed OCTA changes in patients with ACS and we meta-analyzed, where possible, data reporting retinal microvasculature using OCTA metrics, namely superficial vascular plexus (SVP), deep vascular plexus (DVP), and FAZ. We found no statistically significant difference in the OCTA metrics between patients with ACS and healthy participants.

Apart from coronary atherothrombosis, microvascular injury seems to be present in ACS and remains a challenge in cardiovascular medicine. Clinical outcomes, prognosis, and cardiac remodeling in ACS can be explained by the underlying pathophysiological mechanisms. Microcirculation resistance and microvascular damage have a cardinal role in the pathogenesis of ACS [27,28]. CMD refers to the structural and functional remodeling of the microcirculation due to both coronary microcirculation and myocardium changes and can be evaluated by both invasive and non-invasive modalities [29]. CMD can be present in up to 65% of patients with STEMI and has been associated with myocardial damage and adverse prognosis post myocardial infarction (MI). Coronary microvascular obstruction (CMVO) is a term to describe the lack of reperfusion of coronary microcirculation post MI despite the restoration of epicardial vessel patency. Its pathogenesis is based on CMD, ischemic injury, distal embolization, ischemia-reperfusion injury, genetic polymorphisms, ischemic pre-conditioning, and inflammation [28]. CMVO is associated with poor survival, heart failure (HF), and left ventricular remodeling, and can be assessed by cardiac magnetic resonance (CMR). Invasive microvascular resistance is expressed by the included index of microcirculatory resistance (IMR), hyperemic microvascular resistance (HMR), and zero-flow pressure (Pzf). High values of MVO and IMR (>40) are related to no scar regression post MI [27,29]. According to De Vita et al. [30], enhanced coronary microvascular constriction and impaired coronary microvascular dilatation are present in patients with NSTEMI. Despite the small number of participants, this study underlies microvascular dysfunction in myocardial ischemia.

Since the retinal microcirculation has neither anastomoses nor capillary sphincters, it is thus considered an end-arterial system. The higher permeability of retinal vessels in combination with the vulnerability of retinal endothelium to oxidative stress, predisposes the retina to microvasculature dysfunction. Changes in the retinal microvascular network have been associated with a number of cardiovascular risks, like hypertension, DM, and dyslipidemia, and with cerebrovascular disease and chronic kidney disease as well [31]. In 2006, Witt et al. [32] showed a relationship between retinal microvascular changes, quantified by a computer-based technique used for digitalization and analysis of retinal photographs, and ischemic heart disease (IHD) mortality. In this case-control study of 126 IHD patients and 528 healthy participants, IHD mortality was positively associated with abnormal bifurcation optimality and negatively associated with simple arteriolar tortuosity. Since then, a plethora of studies have quantitatively described retinal microvascular alterations and dysfunction in patients with CAD [10,12].

OCTA is a non-invasive, useful imaging modality to measure vessel density (VD) and perfusion in the macula, the optic disc, and the choroid, and investigates alterations of blood flow in these areas. Thus, it gives a detailed evaluation of the microvascular network and segmentation of the FAZ, as well as the superficial, deep, and choroidal plexus [33]. During the past few years, a number of systematic reviews and meta-analyses have been published that elucidate the role of OCTA in ophthalmic conditions, their diagnosis, and classification such as diabetic retinopathy (DR), glaucoma, retinal vasculitis, and myopic choroidal neovascularization [34-37]. A number of recent systematic reviews and meta-analyses have also introduced the invaluable role of OCTA changes in retinal microvascular network as potential novel biomarkers in diseases such as hypertension, multiple sclerosis, Alzheimer’s disease, and Parkinson’s disease [38-41].

OCTA's contribution to quantifying changes in retinal microcirculation in CAD has proven invaluable [12]. The majority of the studies included in the analysis, introduced and measured modifications in the FAZ area, SVP, and DVP. In this regard, OCTA changes may also be associated with CAD severity and prognosis. Sideri et al. [11] showed that left ventricular ejection fraction (LVEF) was significantly associated with the perifoveal area of SCP (β coefficient = 0.06; 95% CI: 0.02 - 0.10, p= 0.006), the perifoveal area of the DCP (β coefficient = 0.10; 95% CI: 0.01 - 0.18, p = 0.026), and the CC layer (β coefficient = 0.28; 95% CI: 0.11 - 0.45, p = 0.002). Undoubtedly, LVEF has been described in CMVO [28]. Wang et al. [9] suggested that changes in retinal and choroidal microvasculature could reveal high-risk CAD patients, despite the early stage of the disease. Alterations in SCP and DCP VD, and choroidal flow were negatively correlated with the Gensini score, which evaluates CAD severity. The cardiovascular risk profile is also reflected and predicted by OCTA alterations, as suggested by Arnould et al. [16], who found a correlation between inner vessel density of the SCP and the Global Registry of Acute Coronary Events (GRACE) and Reduction of Atherothrombosis for Continued Health (REACH) scores (Spearman r = -0.33, p < 0.001 and r = -0.49, p < 0.001, respectively) in ACS patients. Both scores stratify the risk of recurrent cardiovascular events and mortality in CAD patients.

Another cross-sectional study, conducted by Ay et al. [24], analyzed OCTA metrics in CAD patients based on the Synergy Between Percutaneous Coronary Intervention (PCI) with Taxus and Cardiac Surgery (SYNTAX) score (SS) (SS-I, cut-off score 12, and SS-II), which defines the degree of CAD related atherosclerosis and predicts long-term mortality post coronary artery bypass grafting (CABG, cut off score 25.1) or PCI (cut-off score 28.5). SS-I patients, regardless of their score, had insignificantly decreased VD within a 300 μm wide region of the FAZ (FD-300), SCP, and DCP VD compared to healthy participants. In contrast, patients with SS-II with PCI ≥ 28.5 had significantly reduced whole and parafoveal SCP VD, and FD-300 (p = 0.034 and p = 0.009, p = 0.019, respectively). The same trend in the aforementioned metrics at a statistically significant level was followed by the SS-II CABG group. In the same study, FAZ area and perimeter did not differ between ACS patients and controls (p > 0.7), whereas FD-300 was found to decrease, a result marginally not statistically significant (p = 0.061). It needs to be denoted that both ACS and chronic coronary syndrome (CCS) groups were included. Therefore, changes in OCTA microvascular measurements indicate CMD in patients with CAD, and may also be observed in ACS patients. Yet, no studies have explored the sensitivity and specificity of OCTA for assessing retinal microvascular dysfunction in ACS.

To the best of our knowledge, this is the first systematic review and meta-analysis evaluating the changes in OCTA parameters in patients with ACS. Apart from one study [24], the quality of the included studies was of medium or low risk of bias. For the analysis, we were able to collect and use data regarding the SVP, DVP, and FAZ. Since these OCTA parameters were variably expressed, we used the SMD of the metrics of interest to overcome it. Yet, there are some limitations to our review. Firstly, we are unable to draw any causal inferences, as all the included studies were cross-sectional. Three studies were also excluded from the analysis [16,22,23]; since there was no control group and in one study [23], we could only calculate mean and SD by approximation and we excluded it from the analysis. Hence, the limited number of the included studies and the small number of eyes assessed in each study could also denote a limitation of our study and rendered meta-regression analysis as well as any sensitivity analysis not feasible.

Additionally, we did not have enough data from other metrics to pool together, such as the choriocapillaris flow area and density, FAZ perimeter, and FD-300. It is also important to consider certain characteristics of the studies that may represent methodological heterogeneity. The inclusion criteria varied among studies, different types of OCT machines, OCTA software, and segmentation methods were used in the selected studies, and the method of eye selection differed among the included studies. Lastly, ACS is an umbrella term for the signs and symptoms of myocardial ischemia and includes STEMI, NSTEMI, and unstable angina [42]. Only one study [24] included in our meta-analysis grouped patients in different types of ACS, yet it did not further include and analyze data for each of them. Thus, subgroup analysis was not conducted.

## Conclusions

This systematic review investigated studies that reported and assessed differences in OCTA metrics between ACS patients and healthy individuals. The analysis of the data suggests that SVP, DVP, and FAZ did not differ significantly between STEMI patients and healthy participants. Hence, the tendency of the eyes of patients with CMD to have decreased vessel density in retinal vascular plexuses was not confirmed. However, the number of OCTA parameters and the number of studies included in the meta-analysis were small. In the future, more prospective studies with larger sample sizes need to be conducted to assess this association and to understand how OCTA metrics could potentially be used as biomarkers of cardiac ischemia and its progression, especially in the microcirculation.
